# Novel Challenges and Opportunities for Anesthesia and Perioperative Care in Microvascular Flap Surgery: A Narrative Review

**DOI:** 10.3390/clinpract14050172

**Published:** 2024-10-18

**Authors:** Aleksi Matias Ojuva, Rihards Peteris Rocans, Janis Zarins, Evita Bine, Insana Mahauri, Simona Donina, Biruta Mamaja, Indulis Vanags

**Affiliations:** 1 Department of Anaesthesia and Intensive Care, Riga Stradiņš University, Dzirciema Street 16, LV-1007 Riga, Latvia; rihardspeteris.rocans@rsu.lv (R.P.R.); insana.mahauri@rsu.lv (I.M.); biruta.mamaja@aslimnica.lv (B.M.); indulis.vanags@rsu.lv (I.V.); 2 Department of Internal Diseases, South Karelia Central Hospital, Valto Kakelan Street 1, 53130 Lappeenranta, Finland; 3 Intensive Care Clinic, Riga East Clinical University Hospital, Hipokrata Street 2, LV-1079 Riga, Latvia; evitabine@rsu.edu.lv; 4 Department of Hand and Plastic Surgery, Microsurgery Centre of Latvia, Brivibas Street 410, LV-1024 Riga, Latvia; janis.zarins@mcl.lv; 5 Baltic Biomaterials Centre of Excellence, Headquarters at Riga Technical University, Pulka Street 3, LV-1007 Riga, Latvia; 6 Institute of Microbiology and Virology, Riga Stradins University, Ratsupites Street 5, LV-1067 Riga, Latvia; donsimon@inbox.lv; 7 Outpatient Department, Riga East Clinical University Hospital, Hipokrata Street 4, LV-1079 Riga, Latvia

**Keywords:** anesthesia, perioperative care, microvascular flap complications, reconstructive surgery

## Abstract

Complex microvascular techniques and in-depth knowledge of blood rheology and microanastomosis function are required for success in microvascular flap surgery. Substantial progress has been achieved in preventing complications, but the rate of flap loss is still significant and can have significant adverse effects on the patient. Flap thrombosis, flap hematoma, and flap loss are the most frequent and severe major surgical complications. Advances in understanding the pathophysiology of different flap complications, the use of preoperative risk assessment and new treatment concepts could improve the perioperative care of microvascular flap surgery patients. Our aim was to outline novel avenues for best practice and provide an outlook for further research of anesthesia and perioperative care concepts in microvascular flap surgery.

## 1. Introduction

**Figure 1:**
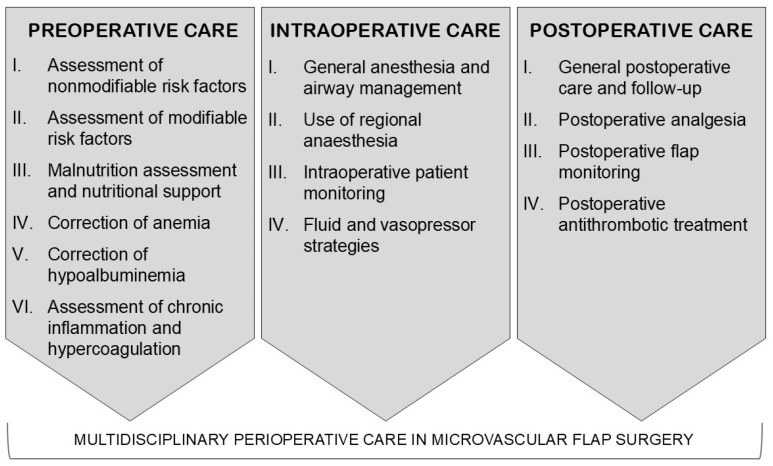
Suggested multidisciplinary team-based approach framework for perioperative care in microvascular flap surgery.

Microvascular flap surgery has secured its place in reconstructive surgery as an important technique to achieve the closure of various tissue defects [1,2,3]. The defect that requires correction can be caused by different etiologies, including trauma, oncology, chronic infection, or wounds of multiple etiologies [1]. The use of free flaps, in contrast to conventional surgical techniques, offers a wider range of donor sites, resulting in improved flap characteristics, such as size, function, tissue components, and form [2]. The use of free flaps also gives the benefit of earlier mobilization, reduced hospital stay and costs [2]. Despite good progress in surgical techniques and reduction of complication rates, flap loss remains a challenge in perioperative care for both surgeons and anesthesiologists. The main complication groups are true flap loss, minor flap complications, and flap hematoma [1,4]. The complication rate varies among studies, although overall, it is between 3 and 6% [3,5,6,7]. Venous thrombosis is the most common cause of true flap loss, while arterial thrombosis is the most common cause of early true flap loss [8]. Other more minor postoperative complications include infection, partial flap necrosis, postoperative bleeding, seroma, and wound dehiscence [3]. Preoperatively, it is important to evaluate and ensure that the current comorbidities of the patient are controlled. Like in all major surgeries, the cardiac and respiratory condition should be optimal, including control of high blood pressure and high serum glucose level [1]. Multiple recent studies have described the pathophysiology of different flap complications and the use of biomarkers for preoperative risk assessment [8,9,10]. These biomarkers may open new avenues for the improvement of perioperative care. After preoperative preparations, high-quality intraoperative care is equally important and must employ good temperature, pain, and sympathetic control to prevent vasoconstriction [11,12,13,14,15]. Optimizing blood pressure and fluid management to maintain high cardiac output and low systemic vascular resistance is crucial to ensure the success of the surgery [1]. Postoperative antithrombotic treatment should be balanced to avoid both bleeding and thrombotic complications [16]. The issues presented are multifaceted; however, perioperative care in a multidisciplinary team-based approach could improve the results of microvascular flap surgery [17]. The aim of this narrative review is to outline novel avenues for best practice in microvascular flap surgery and provide an outlook for further research on preoperative risk assessment, anesthesia, and perioperative care. This narrative review separately addresses the three main groups of issues in preoperative, intraoperative, and postoperative care (Figure 1).

## 2. Materials and Methods

For this literature review, PubMed, Scopus, and Web of Science databases were used. The search was carried out using the following algorithm and key terms: ‘’preoperative risk’’, ‘’risk factors’’, ‘’biomarkers’’, ‘’fibrinogen’’, ‘’malnutrition’’, ‘’comorbidities’’, ‘’anemia’’, ‘’coagulation’’, ‘’coagulogy assessment’’, ‘’inflammation’’, ‘’regional anesthesia’’, ‘’general anesthesia’’, ‘’analgesia’’, ‘’crystalloid’’, ‘’vasopressor’’, ‘’fluid’’, ‘’monitoring’’, ‘’anticoagulants’’, ‘’antiaggregants’’, ‘’intraoperative care’’, ‘’preoperative care’’, ‘’postoperative care’’, “continuous instrumental free flap monitoring” combined with ‘’free flap failure’’, ‘’true flap loss’’, ‘’minor flap complications’’, ‘’flap complications’’, ‘’free flap thrombosis’’, ‘’free flap surgery’’, or ‘’microvascular flap surgery’’. Articles published from 1 January 2007 to 1 July 2024 were selected and analyzed. The articles were selected for the review based on relevance to daily clinical practice, with an emphasis on the studies reflecting new avenues for predicting risk, providing individualized patient care, and improving outcomes for clinical practice. Upon selection for review, the highest priority was placed on randomized clinical trials, larger observational studies, and the most recent articles.

The review uses the following definitions for complications: true flap loss is defined as the impairment of the flap blood flow due to venous or arterial anastomosis dysfunction or thrombosis leading to congestion or ischemia and complete loss of the free transposed flap; flap hematoma is defined as the presence of a postoperative hematoma adjacent to the flap recipient site without interfering with the flap blood supply either due to surgical causes or insufficient coagulation function; minor flap complications are defined as the presence of flap recipient or donor site wound infection, slow or difficult flap wound healing, partial or marginal flap necrosis, or difficult healing at the donor site.

## 3. Results and Discussion

### 3.1. Preoperative Assessment of Non-Modifiable Risk Factors

To properly evaluate preoperative risk factors, it is important to assess the patient’s non-modifiable risk factors [3,18]. Evidence on the gender of the patient in relation to the risk of flap failure remains inconclusive. A study by Sanati-Mehrizy et al. that included 1921 patients identified male gender as an independent risk factor for flap failure in all flap types included in the study [18]. However, some studies also found no statistically significant link between male gender and free flap failure [3], and on the other hand, female sex was found to be a risk factor in the case of head and neck free flap failure [10].

Age was not identified as a direct risk factor for flap failure on its own [3]. Despite the increased rate of vascular damage and atherosclerotic changes in older patients, the risk of flap thrombosis is not considered to be higher [3]. Therefore, age should not be a contraindication to microvascular flap surgery [3,18].

The etiology of the defect is non-modifiable at the time of surgery and might also influence the risk of free flap failure. Among the patients in the study conducted by Lese et al., it was found that in elective non-cancer patients, vascular compromise occurred in 3.8% of cases, the cancer group had a compromise rate of 6.9%, and in the trauma group, the rate was even higher at 8.9% [3]. Another study by Bui et al. also found that a history of trauma is linked to increased rates of flap complications [19].

### 3.2. Preoperative Assessment of Comorbidities and Modifiable Risk Factors

Patient comorbidities should be evaluated before surgery to predict and potentially improve outcomes. Comorbidities and factors that have been identified as a risk factor for flap failure include diabetes mellitus [3], peripheral vascular disease [3,8], arterial hypertension [3], coronary heart disease [3], renal failure [6], recent radiotherapy [6], and smoking [18]. However, not all of these were recognized in all studies as independent risk factors, and some of them remain inconclusive. The ASA class should be considered as one of the possible predictors of postoperative complications, with a higher ASA class being a risk factor for flap failure [3]. Smoking was identified as a risk factor for free flap failure in some studies [18], but there were others in which it was not considered an independent risk factor [3,8,20]. Smoking cessation 4–8 weeks before surgery is generally preferred to decrease perioperative morbidity and improve wound healing [20,21].

### 3.3. Preoperative Malnutrition Risk Assessment and Nutritional Support

Nutritional status is an important feature that influences surgical outcome, and malnutrition can be a major risk factor for complications in all surgical patients [22]. Malnutrition has been found to negatively impact wound healing [23], and the same applies to microvascular flap surgery [4,5]. A recent study by Yu et al. showed that a low prognostic nutritional index (PNI) was found to be a risk factor for free flap failure [5]. PNI is calculated using the serum albumin and total lymphocyte count (10 × serum albumin g/dL + 0.005 × total lymphocyte count per microliter) [5]. The study used the cutoff of PNI < 40 to predict the risk of flap complications [5].

In another study, the Controlling Nutritional Status Score (CONUT) was used to indicate the risk of malnutrition [4]. An increased preoperative CONUT score was found to reliably predict flap complications with a score of more than 2 [4]. This score includes some of the same parameters as PNI, such as serum albumin and total lymphocyte count, but also includes total cholesterol [4].

Although evidence can be found for the assessment of the risk of malnutrition, direct evidence for nutritional support in microvascular flap surgery is limited. Upon the writing of this review, only a single clinical trial by Hwang et al. showed that intraoperative enteral feeding during microvascular flap reconstruction in head and neck surgery enhances wound regeneration [24].

Options for nutritional support include oral supplementation, enteral feeding (via a gastric or postpyloric tube), or parenteral feeding [25]. Enteral feeding is generally preferred to parenteral feeding if possible due to better safety, reduced complications, and cost [25]. Providing perioperative nutritional support could benefit patients who are malnourished but also lead to postponed surgery and the additional cost of artificial nutrition [26]. Additionally, it is worth noting that preoperative fasting should be minimized. Clear fluids should be allowed up to 2 h before the beginning of anesthesia, and solid foods up to 6 h prior [12].

In summary, patients with both mild and severe malnutrition may benefit from preoperative nutritional evaluation [4,5] and nutritional support [4], and intraoperative or early postoperative enteral nutrition [12,24] should be encouraged.

### 3.4. Correction of Preoperative Anemia

In microvascular surgery, it is generally accepted that hemoglobin will decrease during the surgery due to hemodilution. In fact, it is traditionally accepted that hemodilution and hyperdynamic circulation can reduce the risk of anastomotic failure [27]. However, both preoperative anemia and intraoperative hematocrit < 30% could be associated with flap failure [28,29]. Furthermore, preoperative anemia is linked to impaired wound healing [30]. A meta-analysis of 4984 patients indicated that preoperative anemia is a possible risk factor for free flap failure [29]. It also indicated that postoperative transfusions are associated with more complications [29]. Perioperatively, the amount of blood loss and blood transfusions should be limited, as they could increase the risk of flap failure and other complications like infection [31]. This, in turn, leads to the importance of identifying preoperative anemia to prevent intraoperative transfusions during surgery [30].

If the cause of anemia is iron deficiency, intravenous or oral iron administration should be considered if the Hb is below 120, depending on the time to the surgery [30,31]. It should be noted that when administering iron treatment, there should be enough time for the correction of the anemia to be effective [31]. For partial correction, two to four weeks, and for full correction, six to eight weeks should be sufficient [31,32]. Intravenous iron corrects iron-deficiency anemia more rapidly than oral iron, but it still requires weeks to improve the results of the complete blood count [32], which must be taken into account during surgical planning.

### 3.5. Correction of Hypoalbuminemia

Albumin is an important protein involved in the regulation of serum osmolality, tissue repair, and systemic inflammation [33]. It acts as the main extracellular scavenger in the interstitium and as an antioxidant, and it also provides amino acids for the synthesis of tissues [33]. In the case of inflammation, capillary permeability increases, which, in turn, leads to an expansion of the interstitial space and an increased distribution of albumin [33]. Furthermore, the half-life of albumin has been shown to decrease the albumin levels in the case of an inflammatory condition [33]. This leads to the conclusion that hypoalbuminemia correlates with inflammation and can be a predictive factor for a negative outcome of the surgery [33,34].

Min, Hong, and Suh found in their study that when the preoperative albumin level was increased by 1 g/dL, the probability of partial flap loss was reduced to less than half [1]. Another study found that preoperative hypoalbuminemia is a negative prognostic factor in patients who have had tumor excision and free flap reconstruction in an advanced stage of head and neck squamous cell carcinoma [9]. A study by da Silva et al. that included 35 patients found that hypoalbuminemia had no impact on the frequency of complications in extremity free flap reconstruction but was associated with a prolonged hospital stay [35]. As mentioned in the previous section, low albumin levels as a marker of malnutrition risk have been associated with an increased risk of free flap complications [4,5,36]. If low albumin levels are measured preoperatively, the cause of hypoalbuminemia should be considered, whether it is due to malnutrition, inflammation, or other causes [33].

Intriguingly, there is evidence that perioperative albumin supplementation may reduce postoperative complications and shorten hospital stays. A study by Xu et al. included 315 patients who underwent oral and maxillofacial tumor resection and reconstruction with free flap. It found that administering 100 mL of 20% albumin intraoperatively and 50 mL every day for 2 postoperative days was associated with fewer local complications and shortened hospital stays. However, this had no effect on systemic complications [37].

### 3.6. Other Preoperative Biomarkers

Fibrinogen plays an important role in tissue inflammation, wound healing, and hemostasis [38]. It is produced mainly by the liver and is also considered an acute-phase protein, so it is also increased by inflammatory reactions [38]. A study by Handschel et al. found that increased preoperative plasma fibrinogen is associated with true flap loss [39]. A study by Drizlionoka et al. involving carriers of a single-nucleotide polymorphism in the gene coding for the gamma chain of fibrinogen showed that these individuals had higher plasma fibrinogen levels and, therefore, also had a higher rate of free flap thrombosis [10].

A recent study found that a high fibrinogen/albumin ratio was associated with decreased microvascular perfusion in patients with ST elevation myocardial infarction who underwent primary percutaneous coronary intervention [40]. A higher fibrinogen-to-albumin ratio has also been associated with poorer prognosis in cancer patients [41,42]. A high fibrinogen-to-albumin ratio could indicate both a state of chronic inflammation [43] and hypercoagulation [44], and both conditions could be hypothesized to be linked to predict flap complications in the future. A study by Vanags et al. showed that Rotational Thromboelastometry could be used in microvascular flap surgery to predict hypercoagulable states and true flap loss in late-surgery trauma cases [45]. When studying only head and neck reconstructions, Stevens et al. found preoperative thrombocytosis to be a strong predictor of flap failure. Whether or not to use antithrombotic treatment in order to prevent this still remains controversial [8].

The neutrophil-to-lymphocyte ratio (NLR) is another biomarker of both inflammation and malnutrition risk [46,47]. A study by Chargi et al. found both the NLR and low skeletal muscle mass are associated with flap complications and increased length of stay [48].

### 3.7. General Anesthesia (GA) and Airway Management

Choosing the appropriate anesthetic technique is dependent on a number of different clinical factors. Anesthesia decision-making is influenced by surgical requirements for the procedure, expected duration, patient comorbidities, postoperative analgesia, and individual anesthesia factors [49]. Generally, due to the multiple surgical sites involved and the positioning of the patient, most microvascular reconstructions are performed under general anesthesia [50]. Specifically, for head and neck reconstruction, airway management for GA can pose specific challenges [12]. In these cases, it is important to carefully examine the patient’s airway before intubation to identify possible tumors, lymphedema, or fibrosis that might have occurred if the patient has received irradiation therapy for cancer [12,51]. Awake fiberoptic intubation is preferred if difficult intubation is expected [12]. Elective tracheostomy prior to microvascular flap surgery has been proposed in extremely difficult airway cases [52].

Regarding the anesthetic agent, there is some evidence supporting the use of Sevoflurane over Propofol. Sevoflurane has been shown to protect the endothelium from ischemia-reperfusion injury in animal models [53]. Sevoflurane creates a lower capillary filtration coefficient when compared to Propofol [11], which may be beneficial in microvascular flap surgery.

After the use of GA, nausea and vomiting prophylaxis must be considered. Specifically for head and neck surgery, postoperative vomiting can cause suture dehiscence, wound infection, and fistula formation [54]. For prophylaxis, patients with increased risk should be identified, and the administration of a combination of antiemetics and corticosteroids intraoperatively should be considered [55]. The choice of anesthetic should also be taken into consideration regarding postoperative nausea and vomiting and favors Propofol over Sevoflurane [12,56].

### 3.8. Use of Regional Anesthesia

The exclusive use of regional anesthesia (RA) instead of GA can help avoid several complications, such as postoperative nausea, airway injury, and respiratory insufficiency. RA could also reduce the number of patients admitted to the ICU postoperatively and improve pain management [57,58]. The use of combined spinal and epidural anesthesia is a considerable option for lower limb reconstruction [57]. Epidural anesthesia can be used exclusively in lower limb reconstruction [59] or be combined with GA as it improves postoperative pain and, according to recent studies, does not increase the risk of flap thrombosis or reduce flap blood flow [14,26]. However, there is also some conflicting evidence on the use of regional anesthesia in microvascular flap surgery [50]. A retrospective study by Jayaram et al. involving 165 patients found that spinal and epidural anesthesia was associated with a higher rate of failure in microvascular free flaps in patients with acute trauma [50]. It has been postulated that the sympathectomy that accompanies regional anesthesia can cause a “steal phenomenon” that diverts blood from the transferred flap to the intact tissue that still has innervation of the autonomic nervous system [50,60]. However, the concept of the ‘’steal phenomenon’’ is applicable to neuraxial anesthesia and may not be applicable to the use of peripheral nerve blocks (PNBs) [61]. The link between neuraxial anesthesia, hemodynamic parameters, and flap complications needs to be further elucidated [50].

Since hemodynamic parameters are generally much less affected when PNBs are used [62], it is reasonable to believe that they are safe and effective for analgesia in microvascular flap surgery [61]. In particular, patients with GA supplemented with peripheral nerve block have no change in the risk of flap complications but have a shorter length of hospital stay [61]. The use of PNBs for the flap donor site [63] as the surgical location receives benefits from RA, much like any other surgical wound [64]. The main PNBs studied in microvascular flap surgery are transversus abdominis plane block for abdominal surgical procedures [65] and superficial cervical plexus block for neck surgery [66], as well as femoral, popliteal, and sciatic nerve blocks for lower extremity surgery [61].

### 3.9. Intraoperative Monitoring and Surgical Aspects

As with all major surgical operations, monitoring the vital parameters of patients is imperative to ensuring successful outcomes and, specifically for microvascular flap surgeries, to prevent true flap loss [67]. Standard basic physiological monitors should be used, such as usual pulse oximetry, electrocardiography, and invasive or non-invasive blood pressure measurement [49,67]. In the case of general anesthesia, additional capnography, end-tidal inhalation anesthetic concentrations, electroencephalography, and, when indicated, neuromuscular blockade monitoring [49] may be used.

Monitoring the intravascular fluid status is also important, as a high volume of perioperative crystalloid infusions has been associated with flap complications [11,12]. Fluids should be administered in a manner that achieves euvolemia, avoiding both hypovolemia and hypervolemia [11,12]. Diuresis, while informative, is not a comprehensive marker of fluid status [68]. More accurate depictions of fluid status can be obtained through the use of a respiratory variation of an arterial line graph, esophageal Doppler technology, and echocardiography monitoring [12,68,69]. It should also be noted that, as with any major surgery, changes in cardiac output are influenced by changes in the depth of anesthesia and surgical simulation [70].

It is mandatory that the patient’s temperature is monitored throughout this type of surgery to maintain normothermia [11,12,13]. Normothermia can be achieved by using warm air covers, warming mattresses, and warming intravenous fluids during the operation [11,12]. Hypothermia has been associated with increased perioperative complications, like postoperative infection rates [11,12,13] and true flap loss [71,72]. A study by Laitman et al. found that hyperthermia also increases the risk of flap complication, and a mean temperature of 36.5 °C is protective against flap complications [73]. The same study found that the temperature interval of 34.5–36.0 °C reduces the risk of flap complications [73]; therefore, the general evidence regarding the optimal intraoperative body temperature remains inconclusive.

Although various macro-hemodynamic parameters can be monitored, there are also multiple ways that the surgeon could check the microcirculatory parameters of the flap intraoperatively. This could also provide information to the anesthesiologist. The main methods of monitoring flap microcirculation include photopletismography [74], the Acland test [75], indocyanine green angiography [76], and implantable Doppler flowmetry [77]. In this regard, the importance of the technical aspects of microvascular flap harvest, flap insertion, and precise blood vessel anastomosis must be emphasized. A meticulous approach to performing the above-mentioned aspects improves free flap outcomes, as this prevents hematoma formation, kinking of the vascular pedicle, and flap loss [78]. Furthermore, diligent instrumental and continuous flap monitoring must be continued postoperatively to optimize microvascular flap survival rates [79].

### 3.10. Fluids, Vasopressors, and Red Blood Cell Transfusions

It is generally accepted that a balanced fluid administration based on the patient’s fluid responsiveness would be preferable [11,14]. The ideal goal would be to maximize cardiac output and tissue oxygenation by aiming toward the peak of the Frank–Starling curve [80] while avoiding flap edema [81]. A study by Dooley et al. about patients with head and neck cancer who underwent free tissue transfer found that higher volumes of intraoperative fluid were associated with an increased rate of both surgical and flap complications [82]. This could be due to the increased susceptibility of the transferred flaps to edema, as they initially do not have lymphatic drainage, and the permeability is increased due to capillary damage [82]. Crystalloid infusion of more than 130 mL/kg per 24 h was suggested by one study to be associated with an increased rate of complications [83]. Another found that patients who received more than 7 L of intraoperative crystalloids had more flap-related complications [81]. The preferred fluid strategy has not yet been elucidated; however, a recent study by Tapia et al. recommended a specific goal-directed fluid management therapy that reduced flap complication rates compared to conventional fluid treatment strategies [69]. It should be noted that the amount of fluid administered is most likely related to the duration of surgery [6]. A duration greater than 18 h was notably influenced by the occurrence of flap failure [6]. Longer duration may cause a longer ischemic period and lead to greater amounts of fluids given to the patient during the procedure [6], which may further exacerbate flap edema alongside increased flap ischemia.

The use of vasoconstrictors in the management of hypotension remains controversial [84,85,86]. In some animal studies, the use of vasopressors has been shown to reduce flap blood flow [87]. Conversely, in multiple studies involving microvascular surgery in humans, the use of vasopressors has not been shown to cause flap complications [82,85,88]. Dobutamine remains an alternative to pure vasoconstrictors and has been shown to improve flap blood flow [7,12,89]. Furthermore, norepinephrine has been shown to improve free flap blood flow, indicating that both of these vasoconstrictors are safe for microvascular flap surgery [90,91]. Conversely, a study by Chang found that the use of vasopressors increased the rate of arterial flap complications and the need for reoperation but did not increase the rate of true flap loss [84].

**Figure 2:**
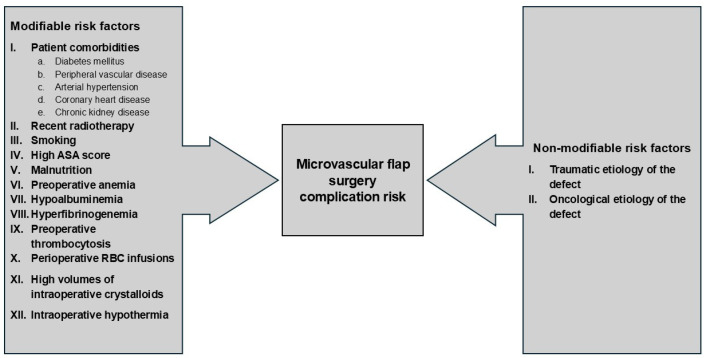
Preoperative and intraoperative risk factors for postoperative complications in microvascular flap surgery. Abbreviations: ASA—American Society of Anesthesiologists; RBC—red blood cell.

The infusion of red blood cells has been associated with an increased rate of flap complications and general complications [11,12,92,93], and this may be due to the immunomodulatory effect of the transfused blood product [12,94]. A link between red blood cell infusions and wound infections has also been demonstrated [95,96]. Therefore, some authors propose a restrictive use of intraoperative RBC transfusions [12,13]. On the contrary, a study by Kim et al. used multivariate analysis to show that the lowest perioperative Hb level and age were significant predictors of flap failure, and the presence of perioperative blood transfusion was not associated with the risk of flap complications [97]. The overall preoperative and intraoperative risk factors have been summarized in Figure 2.

### 3.11. General Postoperative Care Principles and Postoperative Follow-Up

Optimal care for microvascular flap surgery patients does not end in the operating room. Postoperatively, the assignment of proper vitals monitoring and monitoring flap viability is of utmost importance. Regarding the location of postoperative care, the consensus within recent studies seems to point towards not routinely admitting patients to the ICU for postoperative care [12,98]. They have shown that there has been no difference in flaps lost due to complications between admissions to the specialist ward and admissions to the ICU [12,98,99]. There was also no difference in ICU readmissions, and general morbidity was not shown to increase in patients cared for in a specialist ward rather than in the ICU [12,98]. For example, a study by Yalamanchi et al. involving 338 patients did not show any differences in flap survival, reoperation, readmission, and complications postoperatively when comparing admission to the ICU and the non-ICU setting [99]. The findings of a meta-analysis by Mashrah et al. were also in line with this consensus [98].

If postoperative care is provided in the ICU, deep sedation and artificial ventilation should be avoided, as this could lead to a prolonged weaning time from mechanical ventilation and increase the risk of pneumonia [12,98]. Avoiding postoperative ICU admissions could also relieve ICU beds for other more critically ill patients and is likely more cost-effective [99,100].

Wherever the patient is admitted for postoperative care and monitoring, it should be performed by adequately trained nursing staff and in a controlled environment. As with all major surgeries, it should include at least vital signs such as heart rate, pulse oximetry, blood pressure measurement, core temperature, blood glucose, diuresis, drainage measurement, and fluid administration [101]. The monitoring should include a physical examination of the flap every hour. The color, capillary refill, tissue temperature, turgor, pinprick test, and Doppler signals should be examined [98]. In the case of flaps that are poorly accessible, there may be a higher benefit from an implantable Doppler [98] or other available instrumental tools.

### 3.12. Postoperative Pain Control

Many different analgesic medications have shown benefits in microvascular flap surgeries [12,102]. It is important for the patient’s postoperative experience to effectively prevent pain for the patient during the postoperative period, and clinicians should depend on multimodal analgesia (MMA) by combining opioids, non-opioid analgesics, and RA, acting through multiple components of the pain pathway [12].

MMA combining non-steroidal anti-inflammatory drugs (NSAIDs), paracetamol, and gabapentin with opioid analgesia has been shown to be safe and effective in postoperative analgesia in microvascular flap surgery patients [15,103]. Compared to standard opioid analgesia, the MMA group had a lower rate of partial flap loss [15]. Another study included ketamine in their multimodal analgesia protocol and found that with gabapentin it proved to be a viable option that could be considered to reduce opioid use [103]. Additionally, ketamine may have a more optimal side effect profile with respect to hemodynamics than opioids, which could affect flap viability [103]. There is also evidence that the use of antiemetic and analgesics could be reduced with a single dose of gabapentin before the surgery [11,104]. It has been suggested that ketorolac may reduce thrombotic complications in microvascular flaps of the lower extremities [105]. However, the general contraindications and side effects of NSAIDs must also be taken into account for all patients [106].

### 3.13. Postoperative Antithrombotic Treatment

The most common cause of flap failure is thrombosis at the anastomotic site; therefore, it is natural to consider using antithrombotic therapies to mitigate this risk. However, the use of anticoagulants also carries an increased risk of bleeding and the formation of hematoma [16]. Many patients undergoing microvascular flap surgery have cancer as an indication for reconstruction, which also increases the importance of good venous thromboembolism prophylaxis to prevent serious complications, such as deep vein thrombosis and pulmonary embolism [12,16]. However, it should be remembered that the pathogenesis of arterial thrombosis is related to endothelial damage, which leads to platelet aggregation, while venous thrombosis is due to fibrin clotting [16]. Furthermore, the risk of flap thrombosis and true flap loss is highest in the first 48 h after surgery and is significantly reduced after 72 h [16].

Commonly used anticoagulants and antiaggregants include unfractionated heparin (UFH), low molecular weight heparin (LMWH), aspirin, and dextran [7,11]. Dextran, a polysaccharide volume expander with antithrombotic properties, is no longer widely considered useful in microvascular flap surgery [107,108,109]. A meta-analysis by Dawoud et al. compiled eight studies on anticoagulation in head and neck reconstruction surgery and evaluated the use of UFH and LMWH [16]. The study found that UFH consistently increased the relative risk of flap hematoma and bleeding when compared to the control and LMWH [16]. LMWH can be used prophylactically, but additional therapeutic dose anticoagulation is not beneficial [16].

Another study that included 843 patients showed that the flap failure rate was not affected by postoperative antiplatelet treatment, intraoperative heparin bolus, or tPA [7]. Multiple studies have shown an increased risk of bleeding and hematoma formation and a lack of improvement in the risk of true flap loss with regard to UFH [110] and aspirin [111,112]. Conversely, a study by Rothweiler et al. that included 178 free flap surgeries found a decreased true flap loss risk using a combined anticoagulation regimen of aspirin 300 mg intraoperatively followed by aspirin 100 mg/day and an intraoperative bolus of UFH 20 IU/kg followed by UFH 500 IU/h with no APTT target value [113]. A study by Karimi et al. proposed a more delicate regimen of aspirin (100 mg/day) for the first 5 postoperative days and enoxaparin 40 mg/day subcutaneously for 3 days [114]. Given the heterogeneity of the evidence, it is clear that the optimal postoperative antithrombotic regimen has yet to be elucidated.

## 4. Areas of Future Research

Despite rapid improvements in this field, multiple knowledge gaps still persist. It has become clear that the factors affecting the complications and success of free flap microvascular reconstructions are highly complex and multifactorial. In general, there is much evidence on different factors associated with complications in microvascular flap surgery. Some studies, however, have quite small sample sizes, making them less reliable for recommendations, and most are retrospective in nature, making them more prone to selection and misclassification bias. The evidence on some factors, such as the assessment of nutritional status, correction of preoperative anemia [28], postoperative analgesia [102,103], and the use of PNB [61,65,66], is clear and generally accepted. In these areas, the main concepts have been agreed upon, yet more studies on their application and specific guidelines are necessary. Preoperatively, albumin supplementation [41], chronic inflammation [47,48], and hypercoagulability assessment [10,45,47] currently lack sufficient data and are highly promising avenues for further research. For intraoperative care, further studies are crucially needed to refine optimal body temperature management [71,72,73], as well as fluid [69,81] and vasopressor [84,85,86] strategies. With regard to postoperative care, continuous instrumental monitoring for free flap transfer is preferable, although the major issue of optimal postoperative antithrombotic regimen [110,111,112,113,114] is yet to be elucidated.

## 5. Conclusions

The main focus of perioperative physicians in microvascular flap surgery is to optimize the patient’s preoperative risks and provide optimal intraoperative and postoperative care. Optimal perioperative care in microvascular flap surgery is increasingly being accepted as a multidisciplinary team-based process. Despite the scientific progress achieved in the field, further exploration is required. This narrative review outlines possible avenues for future research to refine perioperative care and improve outcomes in microvascular flap surgery.

## Data Availability

The data sets used during the current study are available from the corresponding author upon reasonable request.
